# A Model of *Drosophila* Larva Chemotaxis

**DOI:** 10.1371/journal.pcbi.1004606

**Published:** 2015-11-24

**Authors:** Alex Davies, Matthieu Louis, Barbara Webb

**Affiliations:** 1 School of Informatics, University of Edinburgh, Edinburgh, United Kingdom; 2 Centre for Genomic Regulation (CRG), The Barcelona Institute of Science and Technology, Barcelona, Spain; 3 Universitat Pompeu Fabra (UPF), Barcelona, Spain; Northeastern University, UNITED STATES

## Abstract

Detailed observations of larval *Drosophila* chemotaxis have characterised the relationship between the odour gradient and the runs, head casts and turns made by the animal. We use a computational model to test whether hypothesised sensorimotor control mechanisms are sufficient to account for larval behaviour. The model combines three mechanisms based on simple transformations of the recent history of odour intensity at the head location. The first is an increased probability of terminating runs in response to gradually decreasing concentration, the second an increased probability of terminating head casts in response to rapidly increasing concentration, and the third a biasing of run directions up concentration gradients through modulation of small head casts. We show that this model can be tuned to produce behavioural statistics comparable to those reported for the larva, and that this tuning results in similar chemotaxis performance to the larva. We demonstrate that each mechanism can enable odour approach but the combination of mechanisms is most effective, and investigate how these low-level control mechanisms relate to behavioural measures such as the preference indices used to investigate larval learning behaviour in group assays.

## Author Summary

The larvae of the fruitfly are attracted to many odours. We use a computational model in which simulated larvae stop, start and redirect their crawling behaviour in response to their experience of changes in odour. We show that three simple rules for switching between behaviours are sufficient to produce larva-like results in a simulated agent.

## Introduction

It is well established that *Drosophila* larvae perform chemotaxis towards a wide range of odourants (e.g. [1]). Our aim in this paper is to examine what sensorimotor mechanism(s) account for larval chemotaxis, looking for a minimal model that captures observed phenomena. This will allow us to examine the nature of sensory input and its processing, and identify possible key control outputs that are modulated by conditions or experience. In particular, we are interested in connecting models of odour discrimination and learning to the odour experience of the animal as it moves in a gradient.

Many other organisms also exhibit chemotaxis, using a variety of different strategies [2]. The basic forms of orientation mechanism are reviewed in [3]. Bacteria alternate straight swimming and random tumbling, with the probability of switching modulated by the direction of change in chemical intensity [4]. In *C. elegans*, a similar modulation of the frequency of large re-orientations (pirouettes) by the odour gradient is accompanied by a more gradual directed bias of runs towards the odour [5]. Insects such as the silkworm moth that navigate in patchy odour plumes make upwind surges in response to odour encounters, interspersed with zig-zag and casting behaviours [6]. Flies approaching odour sources in relatively still air might do so by alteration of their visuomotor control, to increase straight flight and suppress turning if odour concentration is increasing [7, 8]. Note that none of these strategies requires the use of spatially separated olfactory sensors to obtain instantaneous measurement of the direction of an odour gradient, but rather exploit temporal change due to movement of the animal, movement of the chemosensors, movement of the medium carrying the odour, or a combination of all three. However, in many cases a bilateral arrangement of sensors does make instantaneous assessment of the relative concentration across space possible, and this is sometimes exploited: for example, bees [9] and flies [10, 11] exhibit turning towards the antenna experiencing higher concentration.

*Drosophila* larvae’s olfactory sensors are located at the tip of the head [12, 13]. As larvae have left and right olfactory sensory organs it would seem possible that they could compare between left and right odour concentrations to perform odour taxis. It has been reported that crude unilateral surgical ablation of sensory organs leads to increased turning towards the intact side [14]. However the separation between these sensors is very small and it seems unlikely that the minute instantaneous difference in concentration between left and right could be detected over environmental, sensory and neural noise [15]. Furthermore, using genetic rescue of single olfactory neurons, it has been demonstrated that while bilateral sensory input improves chemotaxis, it is not required [16].

The most salient features of the larva’s movement patterns also seem inconsistent with instantaneous lateral steering. Larval locomotion has two distinct modes [17]. During runs, consistent peristaltic waves cause the larva to move forwards in a relatively straight line (but see below). During turns, unilateral contraction of one side of the body or the other causes the anterior section of the body to sweep from side to side, a behaviour referred to as ‘head casting’. The effective direction of a turn is determined by the casting behaviour ending with the anterior section of the body at an angle to the rest of the body. In this case, when the larva resumes running, it moves off in a new direction with respect to the previous run. As it moves forward, the rear gradually realigns itself with the front. Larvae have been shown to produce run and turn behaviours without the brain [18], suggesting they may have a ‘basic’ locomotion pattern embedded in the ventral nerve cord and motor system, which can be modulated by higher brain areas in response to sensory input.

The most detailed behavioural description of larval *Drosophila* chemotaxis comes from [19]. By using an arena designed to produce a well-defined odour gradient (described in [16]), and fine-grained tracking of individual larvae exploring this environment, the authors were able to decompose larval behaviours based on orientation with respect to the local odour gradient. This analysis revealed that larvae were 1) more likely to stop runs and start head casting when moving down gradient, and 2) more likely to turn (i.e. finish head casting and return to running) towards the direction of higher odour concentration. Similar results have been reported in a study using linear odour gradients [20].

But how do larvae determine when to turn and which direction to turn? Turn initiation is typically preceded by a period of decreasing sensory experience (defined as a normalised derivative of concentration) corresponding to running down the gradient [19]. Turns to the direction of higher concentration are typically preceded by a large spike in sensory perception, corresponding to a head cast in the direction of higher concentration. Given that the direction of a turn (the alteration in direction between two runs) is determined by the direction of the head cast preceding the turn, a large spike in sensory perception could act as a signal to transition from head casting back to forward movement, resulting in turns generally being towards the direction of high concentration [19].

More recently a third factor contributing to odour-directed paths in larvae has been described [21, 22] which has been termed ‘weathervaning’. During runs, the larva’s path tends to be curved slightly but significantly towards the side of higher odour concentration. This behaviour can still be observed for larvae with single, unilateral olfactory receptors, and has been hypothesised to utilise active sensing of the lateral olfactory gradient through low amplitude head casts during runs [21].

In this paper we use an agent based model to determine if these three control mechanisms—initiating head casting when the odour intensity is decreasing, ending head casting when a sharp increase in odour is experienced, and ‘weathervaning’—can be derived from simple perceptual processing; whether they can replicate fine-grained statistics of larval behaviour; whether they are necessary and/or sufficient to produce chemotaxis; and whether they can be used to provide a low-level account for high-level behavioural descriptions such as preference indices.

## Models

### Simulated larva

**Fig 1:**
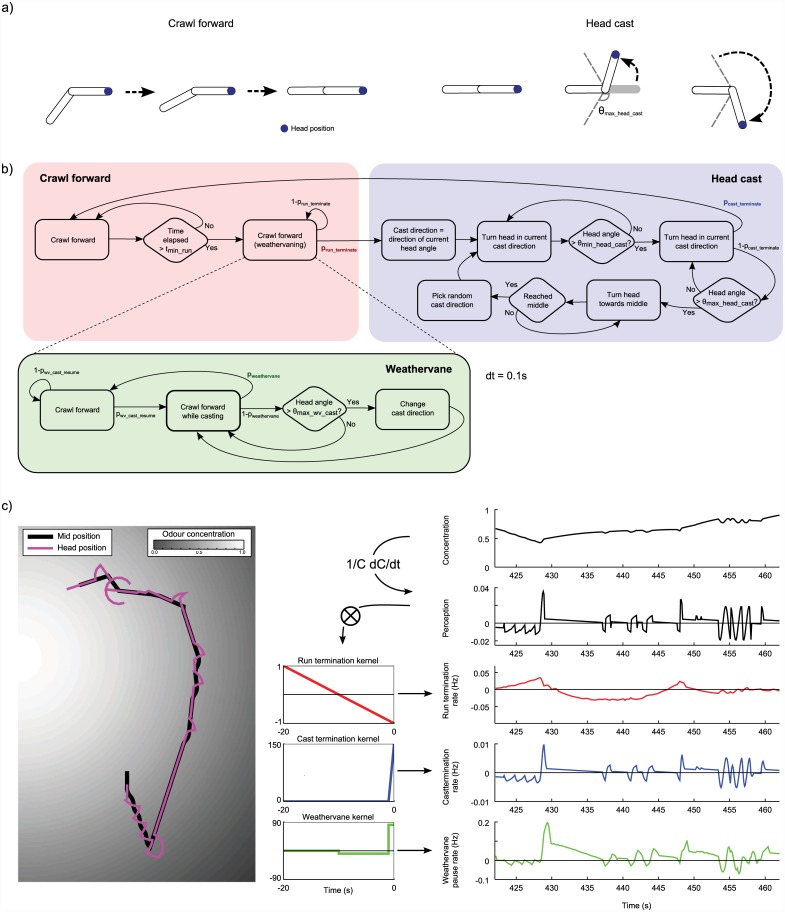
Outline of the simulated larva. **a)** Basic behaviours. The simulated larva can move forward in the direction it is oriented with the body rotating to align (runs); or the head section can rotate back and forth relative to the body (head casts). **b)** State transition diagram illustrating the control of behaviour in the simulated larva. Transitions between running and head casting and pauses in weathervane casting are probabilistic, with the probabilities modulated by the preceding sensory experience. (Note that the wall contact response is not shown, see text). **c)** Perceptual experience of a simulated larva in an odour gradient. A typical body / head trajectory through a Gaussian odour landscape is shown on the left. The concentration, perceptual measure, and behavioural transition rates modulations experienced by the model as it follows this trajectory are shown on the right. The perceptual measure is obtained by taking the normalised rate of change of the odour concentration, while the behavioural transition rate modulations are obtained by convolving the perceptual measure with the associated kernel (shown beside each trace).

We abstract the body of a larva as consisting of two sections of equal length, the head and body, with one articulation between them. The basic larva has two distinct behaviours, runs and head casts (Fig 1a). During a run, the head section moves forward at constant speed *v*_*forward*_. Head orientation remains constant during a run, apart from slight modulation by the weathervane mechanism (see below). The body section is ‘pulled’ behind the head section during runs; when the head body angle is not zero the body section gradually rotates to align with the head section as the larva moves forward. During head casting, the body section remains motionless while the head section rotates from side to side relative to the body section, at speed $\theta_{cast}^{\prime }$. Upon reaching the limit of rotation, *θ*_*max*_*head*_*cast*_, in one direction, head rotation in the opposite direction begins immediately. Head casting may terminate with the head section oriented differently to the body section; this orientation will determine the direction of the following run, and thus the effective size and direction of turns. All our simulations consist of single larva trials, and we therefore do not consider collisions or interactions between larvae.

### Odour environment and perception

The intensity of the odour at any location in the simulation is given by a single value; in this paper we consider only single-odour environments (as used in many behavioural experiments). We use both artificial odour gradients (e.g. a Gaussian distribution of concentration around an odour source), and data taken from measurements of real experimental odour landscapes in which larvae have been tested.

For a simulated larva in a given landscape we use the odour concentration C at the tip of the head section as the input to the larva’s ‘perception’. This perceptual value is generally the only information the simulated larva has about the environment, and all behavioural modulation is based on a limited history of this value.

Following [19], we approximate perception with a rule of the form:

$$\begin{array}{c} \phi =\frac{1}{C}\cdot \frac{dC}{dt} \end{array}$$ (1)

This rule gives the larva access to a measure of the relative rate of change of the odour concentration. When moving up gradient the perceptual value will be positive, and when moving down gradient the value will be negative. Note that this perceptual processing is deliberately simple, intending to capture the hypothesis that a relative rate-of-change perceptual signal is sufficient to allow for larva-like chemotaxis. In reality there must be some level of odour which falls below the perceptual limits of the animal, and some level that entirely saturates the response, but these effects are not included in the current model. We address the issue of more realistic sensory processing in the discussion.

Due to the normalisation in our perception rule, the scale of concentration values in our odour landscapes is arbitrary. Thus for simplicity, unless otherwise noted we normalise the values of all odour environments such that the peak concentration is 1.

### Basic behavioural control

Our starting point for behavioural control of the simulated larva is based directly on the hypothesis proposed by [19], that directed behaviour emerges out of sensory-driven probabilistic transitions between running and head casting that are controlled solely by the recent history of perception (Fig 1b). Specifically, it is assumed that larvae:

1. Increase *p*_*run*_*terminate*_(*t*), the probability of terminating a run and initiating head casting when detecting a gradual decrease in perception value during a run and
2. Increase *p*_*cast*_*terminate*_(*t*), the probability of terminating head casting and resuming running on detecting a sharp increase in perception value.

For our purposes, it is simpler to first define rates of transitions, and convert these into probabilities of transitioning between behaviours on each time step by:

$$\begin{array}{c} p_{run\_terminate}\left(t\right)=r_{run\_terminate}\left(t\right)\cdot dt \end{array}$$ (2)

$$\begin{array}{c} p_{cast\_terminate}\left(t\right)=r_{cast\_terminate}\left(t\right)\cdot dt \end{array}$$ (3)

Note that simulations proceed in discrete timesteps of length *dt* = 0.1*s*.

We can now define our control problem as converting the larva’s perceptual history (the only information it has available to it) into these transition rates. We do this by defining a kernel for each transition, and obtaining a rate of transitions by convolving the perceptual history with the appropriate kernel (i.e. element-wise multiplying and then summing). As larvae make transitions from runs to turns even in the absence of odour stimuli, we further include a base rate of making a transition regardless of the perceptual history.

$$\begin{array}{c} r_{run\_terminate}\left(t\right)=r_{run\_terminate\_base}+\sum_{t^{\prime }=0}^{t_{run\_kernel}}\phi \left(t-t^{\prime }\right)\cdot k_{run\_terminate}\left(-t^{\prime }\right) \end{array}$$ (4)

$$\begin{array}{c} r_{cast\_terminate}\left(t\right)=r_{cast\_terminate\_base}+\sum_{t^{\prime }=0}^{t_{cast\_kernel}}\phi \left(t-t^{\prime }\right)\cdot k_{cast\_terminate}\left(-t^{\prime }\right) \end{array}$$ (5)

To encode the desired behavioural controls into our model, we use simple linear kernels which resemble the average perceptual history preceding behavioural transitions in real larvae, as reported in [19]. Thus the kernel for run termination takes the form of a gradual negative slope, while the kernel for cast termination is a steeper positive slope with a similar duration to a single head cast (see depictions in Fig 1c). Note that both transition rates are continuously calculated regardless of behavioural state, but only affect behaviour when the larva is in the corresponding state. The output of the perception rule *ϕ* and the control rules *r*_*run*_*terminate*_(*t*) and *r*_*cast*_*terminate*_(*t*) for a section of simulated larva paths are shown in Fig 1c.

### Extensions to behavioural control

Initial simulations using the control outlined above raised a number of issues leading to the following modifications to the control scheme.

#### Transition on outward casts only

When a simulated larva head casts towards a side with lower odour concentration, the probability of terminating the head cast (and transitioning back to forward crawling) is low, due to the decreasing odour value. However, when casting back towards the centre after a head cast to the low-odour side, the probability of terminating the head cast rises, making it likely for the cast to terminate before the head has actually returned to the centre. This means that when the run is resumed, the larva has effectively made a turn towards the side of lower odour. To reduce this effect, we altered the behavioural control so that transitions from head casting to running could only occur during the ‘outward’ phase of the head cast, and only when the head cast has reached a minimum magnitude, *θ*_*min*_*head*_*cast*_. One side effect of this change is that in our model head casting is always followed by a turn, i.e. our model never head casts and then continues in its original direction. Larvae do sometimes head cast and then resume their runs in the same direction, however, such events make no difference to the larva’s trajectory, and were excluded from analysis of the larval data to which we later compare our simulation’s behaviour [19].

#### Enable repeated casts to one side

Real larvae show significant instances of consecutive head casts to the same side. To allow our model to match this behaviour, we altered the head casting behaviour such that when the head crosses the centreline, the simulated larva has a 50% probability of reversing the current head rotation direction so as to cast back in the same direction again.

#### Minimum run duration

With the basic behavioural control scheme described above, it is possible for larvae to transition from head casting to running, and then immediately transition back to head casting. This tends to distort the proportions of apparent consecutive head casts. To prevent this, we added a period of length *t*_*min*_*run*_ following transitions to running during which a transition back to head casting cannot occur.

#### Weathervaning

The basic controller produces straight runs only, but it is clear that larval behaviour contains curved runs, and as discussed in the introduction, it has been shown that these tend to curve the path towards the side of higher concentration. We therefore introduced a weathervaning mechanism to the simulated larva, which gradually reorients it towards the odour source during runs. We follow the hypothesis from [21] that larvae sense lateral concentration differences during runs via low amplitude head casts, and use this information to bias the direction of their runs towards the side of higher concentration.

During runs, our simulated larva continuously makes small casts of magnitude *θ*_*max*_*weathervane*_*cast*_ in alternating directions at an angular speed of $\theta_{weathervane\_cast}^{\prime }$, while still moving forwards in the direction of the head section. We refer to these casts as ‘weathervane casts’ to distinguish them from the larger casts made when running has stopped. The model probabilistically transfers from weathervane casting to simple forward crawling at rate *r*_*weathervane*_*cast*_*terminate*_. If this transition to simple forward crawling happens when the head is at an angle to the body, the resulting reorientation of the body will lead to a curve in the path. The transition back to weathervane casting also happens probabilistically, at constant rate *r*_*weathervane*_*cast*_*resume*_.

*r*_*weathervane*_ is calculated in a similar manner to *r*_*run*_*terminate*_ and *r*_*cast*_*terminate*_, with the only difference being the choice of kernel and base transition rate. To produce a weathervaning effect, we want the model to preferentially pause its weathervane casting when the head is angled towards the side of higher odour concentration. Intuitively, this suggests a kernel similar to that used for cast termination, which translates short term rises in perceptual value to high cast termination rates. However, for weathervaning there is an additional complication—the perceptual signal results from a combination of both forward motion and lateral casting. We therefore use a kernel which looks for an increase in perceptual signal relative to a longer term average, as displayed in Fig 1c. When convolved with the perceptual history, this kernel calculates a value proportional to the mean perception of the last *t*_*weathervane*_*short*_*average*_ seconds minus the mean perception of the last *t*_*weathervane*_*long*_*average*_ seconds.

#### Biased first cast direction

[19] show that around 70% of the time, a larva’s first head-cast after terminating a run is towards the direction of higher odour concentration. Our basic model casts left or right with equal probability, and so does not capture this phenomenon.

We therefore altered the model such that the direction of the first head cast after terminating a run is determined by the current angle of the head (which is constantly changing due to to the weathervaning mechanism introduced above); if the head is to the left of the mid-line the first cast will be to the left, while if the head is to the right of the mid-line the first cast will be to the right. As weathervaning tends to lead to the larva curving towards the side of higher odour concentration, this should lead to a bias in first head casts to the direction of higher odour concentration.

#### Wall contact

To be able to compare our simulated behaviour with standard test situations for the larva, we need to test it in delimited areas, and hence require a mechanism to determine how simulated larvae will behave when they come into contact with a wall. Collision detection occurs when the head point of the larva coincides with a defined wall location in the environment. A collision while running results in a small turn of the head section in the direction away from the wall. A collision during a head cast leads to a switch in head casting direction. The effective result is that the simulated larvae will run along a wall until it transitions to head casting, and may then end its head casting in a direction taking it away from the wall. Although the behaviour of real larvae when encountering obstacles has not been analysed in detail, this response of the simulated larva is broadly similar to our observations of real larvae.

The resulting complete controller is illustrated in the form of a state transition diagram in Fig 1b.

### Parameter setting

Twenty parameters need to be set for this model. Some can be taken from available data, but the appropriate values for others were less clear. We discuss here how we chose each of our parameters, with the final values used to generate the results in this paper summarised in table 1.

**Table 1:** Parameters.

Parameter	Value	Description
*dt*	0.1*s*	Simulation timestep
*v*_*forward*_	1*mm*/*s*	Speed of forward crawling
*t*_*min*_*run*_	1*s*	Minimum run duration
*θ*_*max*_*head*_*cast*_	120°	Head cast range
*θ*_*min*_*head*_*cast*_	37°	Minimum angle for head cast termination
$\theta_{cast}^{\prime }$	240°/*s*	Rotational speed of head casts
*θ*_*max*_*weathervane*_*cast*_	20°	Weathervane cast range
$\theta_{weathervane\_cast}^{\prime }$	60°/*s*	Rotational speed of weathervane casts
*r*_*run*_*termination*_*base*_	0.148/*s*	Run termination base rate
*k*_*run*_*termnation*_*start*_	2	Run termination kernel start value
*k*_*run*_*termnation*_*end*_	−2	Run termination kernel end value
*t*_*run*_*termnation*_	20*s*	Run termination duration
*r*_*cast*_*termination*_*base*_	2/*s*	Cast termination base rate
*k*_*cast*_*termination*_*start*_	0	Cast termination kernel start value
*k*_*cast*_*termination*_*end*_	150	Cast termination kernel end value
*t*_*cast*_*termination*_	0.5*s*	Cast termination duration
*r*_*weathervane*_*cast*_*termination*_*base*_	2/*s*	Weathervane cast termination base rate
*r*_*weathervane*_*cast*_*resume*_	1/*s*	Weathervane cast resumption rate
*t*_*weathervane*_*short*_*average*_	1*s*	Weathervane kernel short average duration
*t*_*weathervane*_*long*_*average*_	10*s*	Weathervane kernel long average duration
*k*_*weathervane*_*mult*_	30	Weathervane kernel multiplicitive factor

We take the forward movement speed *v*_*forward*_ = 1*mm*/*s* from figure 2a in [19]. From analysis of paths of larvae in a no-odour environment we estimate the base rate of transitions from runs to turns at *r*_*run*_*termination*_*base*_ = 0.148*s* ^−1^, that is, turns occur on average every 7 seconds. To determine the corresponding base rate for turn to run transitions, we count the proportion of turns which have a single associated head cast. We assume that this gives the probability of transitioning from head casting to forward crawling behaviour during any given head cast (making the implicit assumption that the distribution of number of head casts before a turn can be described by a geometric series). We then divide this probability by the duration of a head cast in our model to give *r*_*cast*_*termination*_*base*_ = 2.0*s* ^−1^, that is, a probability of 0.7 of returning to running after a single head cast.

Inspecting head casts from the no-odour larva data, we found that over 95% of casts did not exceed 120°, and so we set *θ*_*max*_*head*_*cast*_ = 120°. The speed of head casts needs to be fast enough to allow up to 4 head casts within a 5 second window (as seen in the larval data), so has been set to allow for a head cast (out and in) of maximal size in one second: $\theta_{cast}^{\prime }=2*\theta_{max\_head\_cast}$. To make turns effective, head casts should only terminate beyond some minimum angle from the centreline. We use the definition of head casts used to define turns in [19], i.e. *θ*_*min*_*head*_*cast*_ = 37°. The amplitude of weathervane casts was set to *θ*_*max*_*weathervane*_*cast*_ = 20°, matching the size of small head casts shown in figure 8c in [21]. Weathervane cast speed was set to a moderate value, $\theta_{weathervane\_cast}^{\prime }=60^{{}^{\circ}}$.

The final parameters to be set are those defining the lengths and shapes of the kernels. As we only use linear kernels, they can be described with three parameters, the duration and the start and end values. We need a relatively long, negatively sloping kernel for the run termination kernel, and a short, positively sloping kernel for the cast termination kernel. On this basis we found approximate values for the kernels by adjusting until the simulated larva displayed navigation towards the odour source.

We then further adjusted kernel parameters by hand until our model matched larval behaviour across a range of behavioural statistics (see Results). This was achieved through gradual adjustment of parameters and visual inspection of resulting behavioural statistics. Automated optimisation of these parameters would have been possible in theory, however, defining a single optimisation criteria when the goal was to match across several distributions would in itself be a subjective process.

The kernel parameters chosen are reported in Table 1; these values are used throughout unless otherwise noted.

### Analysis methods

To establish a comparison between our model and experimental results from real larvae, we apply a number of metrics from [19] to paths of wild type larvae and simulated larvae. These metrics are constructed from the trajectories of larvae’s head, centroid and tail positions; note that in this analysis no use is made of the internal state of the model. The metrics used are as follows:

- **Body angle**, *α*, the angle of the larva’s body section (measured anticlockwise from the x-axis of the arena)
- **Reorientation speed**, $\frac{d\alpha }{dt}$, the rate at which the larva’s body orientation is changing
- **Head angle**, *θ*, the angle between the larva’s body and head sections
- **Bearing**, *β*, the relative angle between *α* and the local odour gradient, where 0 indicates the larva’s body is aligned with the direction of maximal concentration increase.

We also extract times of turns and head casts from our simulations. These are defined as follows:

- **Turn**, a period during which $\frac{d\alpha }{dt}>12^{{}^{\circ}}/s$. Turns of less than 1s are discarded.
- **Head cast**, a period during which |*θ*| > 37°.

Events are categorised as follows:

- **Turns to left / right.** A turn is classified as a left turn if the change in body angle is positive, and as a right turn if the change in body angle is negative. We assume turns are in the shorter direction, e.g. a turn from 170° to −170° is assumed to be a change in bearing of +20°, not −340°.
- **Turns to high / low.** A turn is classified as ‘to high’ if it is in the direction which decreases bearing *β*. Thus for a larva travelling at a bearing between 1° and 180° a turn to the left counts as ‘to high’, while for a larva travelling at a bearing between −1° and −180° a turn to the right counts as ‘to high’. Note that a turn starting at 10° end ending at −20° is still counted as ‘to high’ under this definition; even though the final bearing is further from up-gradient than the initial bearing the direction of the turn was ‘correct’.
- **Head cast direction.** Head casts are also classified as ‘to high’ or ‘to low’, with a definition matching that for turns.
- **Head casts per turn.** A head cast is classified as ‘belonging’ to a turn if the head cast falls within the 5 seconds preceding the initiation of the turn, or between the end of the previous turn and the initiation of the turn, whichever is the shorter interval.

## Results

### Matching behavioural statistics

Our aim when picking kernel parameters was to produce a simulated larva which matches the behavioural statistics of real larvae (n = 42) chemotaxing in an odour gradient of ethyl butyrate, as reported in [19].

We produce behavioural statistics for the simulated larva as follows using the same odour distribution as measured in [19], in a virtual arena of size 65x100mm. Note that the arena size for the larval experiments was 80x120mm, however an estimate of the odour concentration could not be experimentally made at the outer edges.

We run 500 simulated larvae in this arena, for 300s of simulated time each. Each simulated larva begins the run with random starting orientation, at a random position within a 12mm square centred on the odour source. The run of any simulated larva which touches the edge of the arena is truncated at that point, consistent with the acquisition of experimental data.

**Fig 2:**
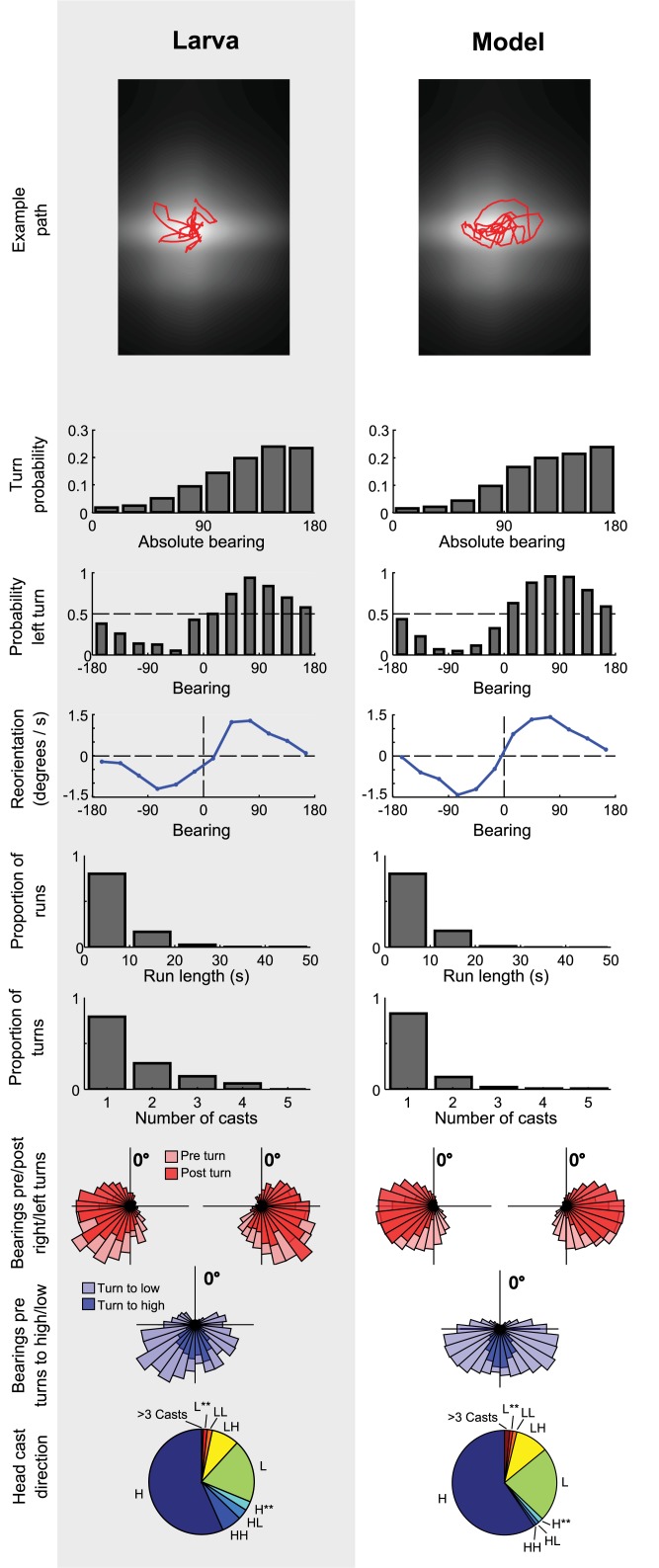
Behavioural statistics for the simulated larva compared to the real larva. Larval data from [19]. See text for full definitions of statistics. The first 3 statistics show that the three modelled chemotaxis mechanisms—biasing of run termination bearing, turn direction, and run curvature—broadly match those of larvae. The remaining statistics demonstrate that the same control mechanisms also produce behaviour comparable to the larva on other metrics, namely distribution of run lengths, number of pre-turn head casts, pre- and post-turn bearings, bearings before turns to the direction of higher and lower odour concentrations, and direction of head casts.

From these simulated trajectories, we calculate various behavioural metrics (as described in the previous section). These are used to produce the behavioural statistics shown in Fig 2.

Note that this process was repeated multiple times as we tuned kernel parameters; we show here only the behavioural statistics obtained with our final set of parameters as reported in Table 1.

Fig 2 shows two sample paths, and the match between real and simulated behavioural statistics. In the top panel of statistics it can be seen that the probability of initiating a turn (an end-run transition) relative to the odour bearing shows the same form as for the real larva. In the second panel, the probability of making a left turn is altered as expected relative to the bearing of the odour, turning left more often if the odour is on the left. In the third panel, the reorientation rate during runs shows a similar dependence on the bearing angle, and similar amplitude.

These comparisons demonstrate that it is possible to choose kernel parameters which result in our model producing very similar behavioural statistics to the larva, on the three metrics which summarise the run termination, cast termination and weathervaning mechanisms. Given that we tuned kernel parameters to match these statistics it is perhaps not surprising that we achieve a close match; nonetheless, it is not trivially obvious that these statistics would be possible to obtain using only linear kernels convolved with the relative rate of change in odour concentration.

The lower panels present comparisons of additional behavioural statistics, which show a number of emergent effects also match well between the model and the larva without additional tuning. The run termination mechanism produces distributions of turn initiation bearings and run lengths similar to the larva. Similarly, the head cast termination mechanism produces turn direction probabilities and numbers of pre-turn head casts comparable to the larva. Both simulated and real larvae tend to be headed away from the odour (>90 degrees) before a turn, and towards it (<90 degrees) after, but with a general undershoot, that is, only a partial correction in orientation. They also both show a similar distribution of bearings that result in correct (to higher concentration) rather than incorrect (to lower concentration) turns, with wrong turns more likely when heading near to 180 degrees away from the odour, a situation which produces the most ambiguous information during head casting.

In the bottom panel, the relative frequency of patterns of head casts towards the direction of higher (H) or lower (L) concentration is shown. Overall the proportions are similar between the larva and the simulations. The larva and the model both show a bias in the direction of the first head cast, with a majority of head cast groups starting with a cast to high. The mechanism by which the larva creates this bias is not yet understood, however, our model produces a similar bias by simply using the angle of its head at the moment of run termination to determine its initial cast direction.

Having set parameters for our model such that it matches the larva on these low level behavioural statistics, we go on to assess the model’s similarity to the larva by comparing its chemotaxis performance to the larva in three different environments.

### Matching near-source chemotaxis

Having tuned our model parameters to qualitatively match low level behavioural statistics of the larva when chemotaxing around a point source of odour, we ask whether this leads to our model quantitatively matching the larva on a higher-level metric, namely the distribution of larvae around the the odour source.

**Fig 3:**
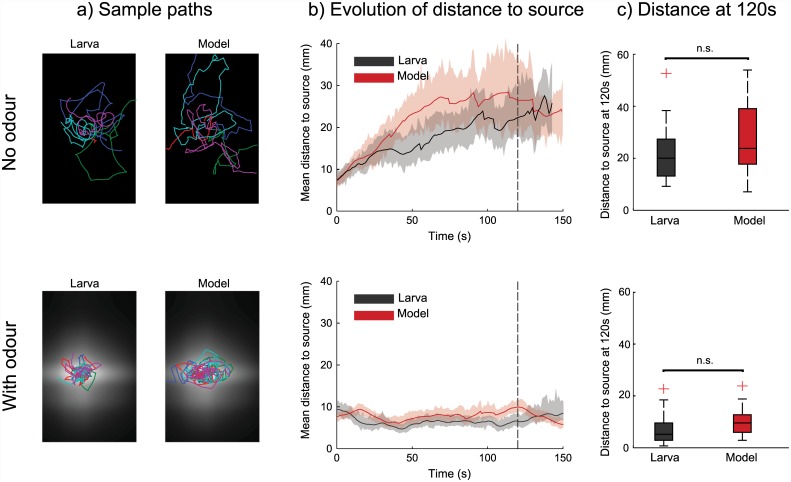
Near-source chemotaxis performance of real and simulated larvae. The first row shows performance of 42 real larvae and simulated larvae with no odour source present, while the second row shows performance of 19 real and simulated larvae with a point odour source of ethyl butyrate, as described in [19]. **a)** Sample paths of real and simulated larvae in this environment. The odour gradient is represented with black representing no odour and white representing maximal odour. **b)** Temporal evolution of the mean distances to the odour peak. Shaded areas show 95% confidence intervals. We use the odour peak position from the ‘odour’ condition to produce this metric for the ‘no odour’ condition. **c)** Boxplot of the distances to the peak after 120s. Boxes show the median and first and third quartiles; whiskers extend to the most extreme non outlier points, where points are considered outliers if they are more than 1.5 times the interquartile distance from the nearest quartile. Distances for the larva and model are not shown to be statistically different for either condition (Mann-Whitney U Test, p>0.05).

Using the data described in the previous section, we computed the distance to the odour source for 42 real and simulated larvae every second for 150s. For comparison, we repeated this process for 19 real and simulated larvae in a ‘no odour’ condition; for the simulated larva this means all behavioural transitions are made at their base rates, with no perceptual modulation. Fig 3 shows sample paths, the temporal evolution of the distance to the source over time, and a snapshot of distances to the source at 120s, for each of these groups.

In the absence of an odour source real larvae gradually disperse; the model performs similarly to the larva in this condition.

With the odour source present, real and simulated larvae both remain located around the source. We use the performance of simulated larvae at 120s as a quantitative measure to determine how closely our model, with parameters tuned to match larvae’s low level behavioural statistics, matches the larva’s chemotaxis performance. The larva and the model’s distances to the source are not significantly different (Mann-Whitney U Test, p>0.05), although this does not provide evidence for the null hypothesis that the medians of the groups are the same. However, using bootstrapping, we can state with a 95% confidence level that the difference in median distance to the source for real and simulated larvae is between -2.4 and 1.9mm, around a single larval body length.

### Matching odour approach

Having confirmed that our simulated larvae show chemotaxis performance at a similar level to real larvae when circling around the odour source, we consider how well they match the larva’s directness of approach to a distant odour source.

For this experiment, we used a second odour gradient of ethyl butyrate (also from [19]), with an odour source centred at one end of a rectangular arena.

Simulations were carried out as above, with a different odour landscape and different starting positions; each simulated larva begins the run at a random position within a 20mm square in line with the odour source on the short axis of the arena and 68mm distant on the long axis. Starting orientations were chosen randomly from a distribution of plus or minus 30 degrees relative to the direction of the odour source. Runs were truncated at the point at which they came within 5mm of the odour peak; runs which did not reach this area were discarded.

For this condition we compare 43 real and simulated larvae. We calculated distances to the odour source every second as above. Following [23], we also use a path tortuosity metric to compare the efficiency of orientation in this landscape; a ‘straightness index’ is assigned to each individual by calculating the ratio of the length of its path to the length of the vector travelled. Paths leading directly to the odour peak will have a straightness index of 1, while paths which follow a less direct route will have a lower straightness index.

**Fig 4:**
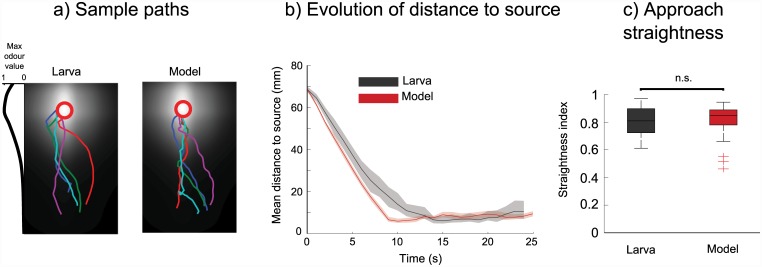
Odour approach behaviour of real and simulated larvae. Performance of 43 real larvae and simulated larvae approaching a point odour source of ethyl butyrate from a distance, as described in [19]. Paths are truncated when crossing the circle indicated around the odour peak. **a)** Sample paths of real and simulated larvae in this environment. **b)** Temporal evolution of the mean distances to the odour peak. Shaded areas show 95% confidence intervals. **c)** Boxplot of path straightness indices (i.e. straight line distance / path length). Straightness indices for the larva and model are not shown to be statistically different (Mann-Whitney U Test, p>0.05).

Fig 4 shows sample paths, the temporal evolution of the distance to the source over time, and boxplots of straightness indices for real and model larvae. We see a good match between the larva and the model’s approach to the odour peak. The larva and the model’s straightness indices are not significantly different (Mann-Whitney U Test, p>0.05), and using bootstrapping, we can state with 95% confidence level that the difference in median straightness index for real and simulated larvae is between -0.11 and 0.03.

### Matching approach in exponential and linear slopes

We next consider the behaviour of the simulated larva in linear vs. exponential odour slopes. We compared the model’s paths to paths of wild type larvae in gradients of isoamyl acetate (landscapes from [16]). Runs were truncated at the point at which they came within a 15mm zone at the peak end of the arena; runs which did not reach this area were discarded. Larvae started within a 20mm square in line with the odour source on the short axis of the arena and 80mm distant on the long axis, facing towards the odour peak. We used the same number of simulated larvae as real larvae in each condition: 20 for exponential, 14 for steep linear, and 11 for shallow linear.

Initial results suggested that with parameters set as described above (to match behaviour in a single source environment of ethyl butyrate), simulated larvae performed significantly worse than the real larvae in this condition. We therefore also tested whether we could improve the performance of the model by scaling (i.e. changing the slope of) the model’s kernels, thereby increasing the strength of the simulated larva’s behavioural biases. We show here results for the model with default parameters, and with a scaling factor of 6 on all kernels.

**Fig 5:**
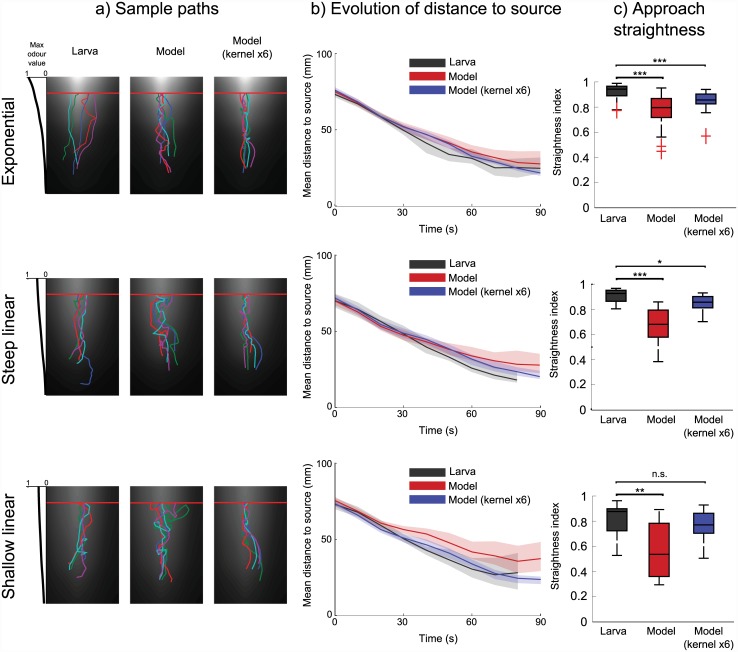
Odour approach behaviour in exponential and linear gradients. Performance of real and simulated larvae in exponential (20 larvae), steep linear (14 larvae), and shallow linear (11 larvae) gradients of isaomyl acetate [16]. A second version of the simulated larva is included for comparison, for which the three behavioural kernels are scaled by a factor of 6. Paths are truncated when crossing the line indicated at the high end of the odour gradient. **a)** Sample paths of real and simulated larvae in these environments. **b)** Temporal evolution of the mean distances to the odour peak. Shaded areas show 95% confidence intervals. **c)** Boxplot of path straightness indices (i.e. straight line distance / path length). The simulated larva shows the same ordering of path straightness indices between conditions, but lower straightness indices overall. Scaling the model’s kernel values produces straightness indices closer to, but not as high as, the larva.

Fig 5 shows sample paths, the temporal evolution of the distance to the source over time, and boxplots of straightness indices for real and model larvae in each environment. These demonstrate that the model (both with normal and scaled kernels) can successfully navigate up both exponential and linear gradients. The ‘straightness index’ shows that the larva more directly approaches the odour peak in an exponential gradient than in a shallow linear gradient; this is also true for the model with either normal or scaled kernels.

However, both the sample paths and the straightness indices highlight the failure of the model with default parameters; simulated larvae in this case follow paths which are clearly more tortuous than the larva. However, by scaling the kernels by a factor of 6, we achieve a much closer match between the model and the larva.

Why do we need to alter our model’s kernel parameters to replicate larval behaviour in this situation? One possibility comes from the difference in the odour used in this condition; our model’s parameters were set to match the behaviour of larvae in an ethyl butyrate gradient, while these gradients were produced using isoamyl acetate. It is possible that the difference in odourants leads to an difference in chemotactic performances for the larva. Alternatively, the differences between model and larval performance in these gradients may be a result of our simplified sensory processing (see discussion). In any case, the fact that the model does chemotax successfully in this environment even with default parameters demonstrates its robustness in a novel odour landscape.

### Relationship to the preference index

A common measure of larval behaviour in odour experiments is the ‘preference index’ [12, 24]. In typical odour-based experiments, a number of larvae are allowed to freely explore a Petri dish (typically 9cm diameter). One or both sides of the dish contain odour sources. At the end of an allotted time period, the number of larvae on each side of the dish (excluding a 1cm centre region) are counted. A preference index is then calculated as:

$$\begin{array}{c} PI=\frac{\#side1-\#side2}{\#total} \end{array}$$ (6)

Preference indices therefore range from 1 to -1, with a positive preference index indicating a preference for side 1, and a negative preference index indicating a preference for side 2. Typical median preference indices for innate chemotaxis range from 0 to close to 1, depending on the odour and concentration used (Schleyer and Reid, pers. comm.).

Unfortunately, the odour environments used in learning experiments are not as carefully controlled and measured as the data we have used for comparisons so far. Odours are presented in the form of a point source, e.g. in a small cup or on a filter paper. Furthermore, the enclosed nature of the Petri dish is likely to lead to non-uniform distribution of the odour, which may also be changing over time. As there are no detailed recordings of odour gradients in these conditions from which we can draw, we assume a very simple odour distribution; a circular Gaussian (*σ* = 30mm) distribution centred on the odour source.

For these trials the simulation arena consists of a circular wall with a radius of 45mm, with an odour source 5mm from the left hand side of the dish. For each odour condition, we ran simulations for 400 individual larvae, each of which was allowed to explore the arena for 5 minutes. Each larva began at a random position on the vertical centre-line of the dish, at a random orientation. At the end of 5 minutes, the position of each larva was recorded. The larvae were split into 20 groups of 20, and for each of these groups a preference index was calculated.

**Fig 6:**
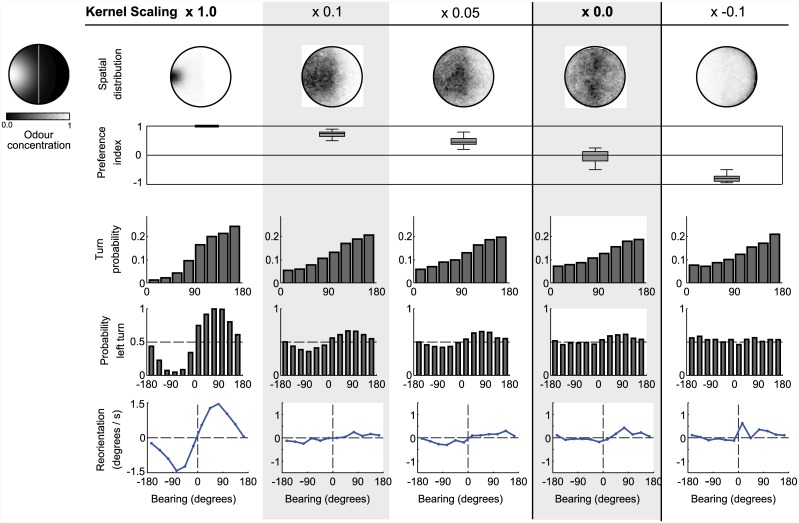
Preference indices for the simulated larvae for kernels of different strengths. Behaviour of 400 simulated larvae over 300s in a choice-assay environment (a Gaussian odour landscape centred on the left side of the dish). Each column corresponds to a different scaling of the kernels used in the model, leading to weaker, abolished, or reversed effects of sensory input on behavioural transitions. The first two rows show the distribution of simulated larvae in the arena, directly through heatmaps and indirectly through the preference index (proportion of larvae on the odour side after 300s). Note that the previously used kernel values (column 1) result in very high chemotaxis performance. The bottom three rows show the main behavioural statistics for these variants of the model (obtained as in Fig 2). Small differences in these behavioural statistics equate to significant differences in spatial distributions in the choice-assay environment.

Initial results, using the parameter settings described above, showed extreme PIs; all simulated larvae ended the 5 minute run on the odour side of the arena, i.e. PI = 1. We therefore proceeded to investigate how scaling (changing the slope of) the model’s kernels, thereby reducing strength of behavioural biases and the efficiency of chemotaxis, changes the observed PIs. Fig 6 shows the distribution of simulated larvae and the corresponding PIs for different kernel scaling factors. Also shown is the the effect of the kernel scaling on the statistics for run termination bearing, turn direction probability, and run reorientation in the original point source environment (as for Fig 2). A scaling of 0.1 produces a strong PI score, and a scaling of 0.05 a score still comparable to larval experiments. With scaling 0, the simulated larva has no behavioural biases and indeed ends up equally distributed across the dish, with PI around 0. Scaling by a negative value produces a negative PIs, that is, apparent repulsion from the odour.

We might expect that our model’s original parameter settings, which we have demonstrated produce behavioural biases of the same magnitude as the larva’s, should produce larva-like PIs. However, we had to considerably reduce kernel scaling, and therefore reduce behavioural biases, to produce moderate PIs. There are several possible explanations for this requirement. First, note that our model has access to a clear odour gradient across the whole arena, unperturbed by noise or sensory thresholds; real larvae may not encounter a consistent gradient far from the source. Alternatively, moderate preference indices could coexist with high behavioural biases if only a fraction of the larvae were displaying those biases, or if all larvae were displaying those biases only a fraction of the time. In preference tests of relatively long duration, this does not seem unlikely. In either case, averaging some combination of strongly biased and unbiased behaviour would result in seeing lower overall behavioural biases for larvae in PI-type experiments, as our model suggests. Finally, there may be effects of group assays such as random reorientations caused by collisions. Unfortunately, data to resolve larval behaviour at an individual level during group assays is not available, restricting our ability to differentiate between these explanations.

### Effect of each control mechanism

Our model combines three sensorimotor mechanisms that appear to operate in the larva to produce chemotaxis:

#### Biased run termination

increased probability of transitioning from running to head casting when the rate-of-change of concentration is gradually decreasing;

#### Biased cast termination

increased probability of transitioning from head casting to running when there is a sharp increase in the rate-of-change of concentration;

#### Weathervaning (wv)

increased probability of pausing ‘weathervane casts’ during running when there is an increase in rate-of-change of concentration relative to the recent average.

With our model, we can investigate the chemotaxis performance of simulated larvae that use only subsets of these mechanisms, i.e. we can compare performance with all three biases, each possible pair of biases, each single bias, or ‘random’ larvae with no biases. Note that it is the modulation of the probabilities of behavioural transitions based on perceptual history which is included or removed, not the behavioural transitions themselves; when a bias is removed the larva makes the associated transition at its base rate, unaffected by the odour gradient. Transitions cannot be removed altogether—a basal rate of run termination is necessary to allow head casting to occur, similarly a basal rate of head cast termination is necessary to allow transitions back to running.

**Fig 7:**
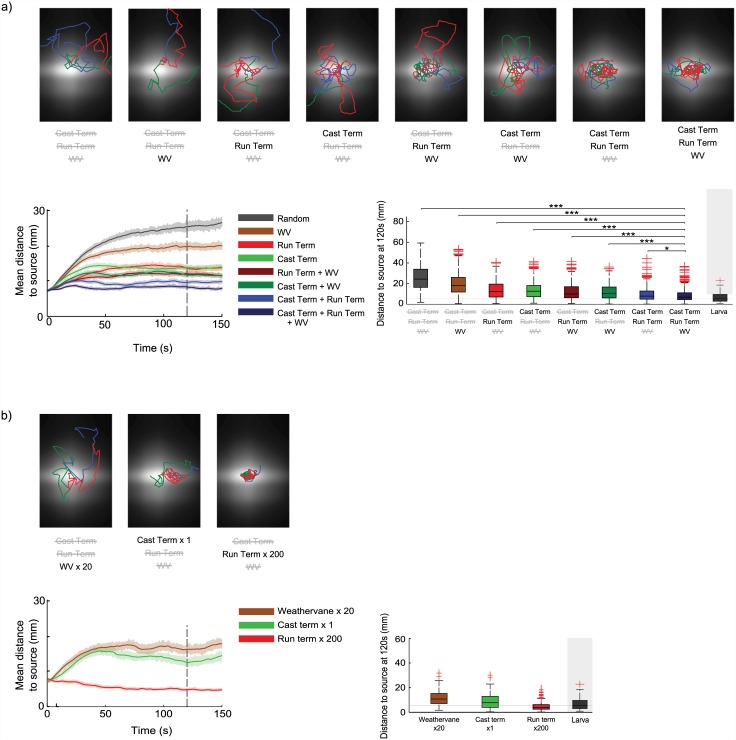
Contribution of behavioural biases. Using an adjusted model in which only some of the three behavioural biases were active, we assess their relative contribution to chemotaxis. **a)** For each combination of biases, we simulated 500 larvae using the same odour environment and protocol as for Figs 2 and 3. We report a selection of sample paths, the temporal evolution of the mean distances to the odour peak, and boxplots of the distances to the peak after 120s. Both run-termination and head cast termination are sufficient on their own to produce effective chemotaxis, but are more effective in combination, and are improved by the addition of weathervaning. The model using all three biases remains more closely clustered around the peak than the model using any other combination of biases (Mann-Whitney U Test, p<0.05 with Bonferri correction). Larva performance in this condition (previously reported in Fig 3) is included for comparison. **b)** Same as in panel a), but with only single active biases, and kernels scaled to give best possible chemotaxis performance. Biased run termination alone can produce very close clustering around the odour source.

We ran 500 simulated larvae for each combination of mechanisms in the single-source condition using the same experimental set up described for Figs 2 and 3. Fig 7a shows sample paths, evolution of the mean distance to the odour source, and boxplots of the mean distances for all larvae in a group 120s after the start of the run.

It can be seen that the combination of all three mechanisms produces the tightest clustering around the odour source. Removing the weathervaning bias leads to only a small reduction in performance, but also eliminates the bias in run curvature observed in the larva (S1 Fig). Either biased cast-termination or biased run-termination on their own, or combined with weathervaning, are sufficient to produce relatively close clustering to the odour source. Weathervaning alone still produces clustering better than random behaviour. To check that these differences are significant, we compare each group to the best performing group (all three mechanisms), and find that all other groups are significantly different (Mann Whitney U Test, p<0.05 with Bonferri correction).

Fig 7a shows the relative contribution to chemotaxis of each of the three mechanisms using the parameters previously set to match larval statistics. However, we can instead ask what chemotaxis performance can be achieved with each single mechanism when parameters are adjusted to maximise chemotaxis instead of match larval behaviour. To this end, we repeated the single mechanisms conditions (i.e. run termination only, cast termination only, or weathervaning only) of the above experiment while varying the scaling (i.e. the slope) of the kernel being considered. This allowed us to determine the kernel parameters which led to the best chemotaxis performance (as defined by mean distance to the odour source after 120s), see S2a Fig. Fig 7b shows sample paths, the evolution of the mean distance to the odour source, and boxplots of the mean distances after 120s, for larvae using single mechanisms with kernels tuned to give maximal performance.

We see that run termination can be highly effective as the only mechanism, to the extent of producing chemotaxis more effective than the larva when the kernel is scaled by 200. It is not clear what constraint might have prevented evolution from finding this solution. By contrast, chemotaxis performance with head cast termination is not improved with kernel scaling above the level required to match larval behaviour. Note that in our model’s implementation of head casting, increasing the slope of the kernel will increase the probability of terminating head casts on the ‘correct’ side, but will also increase the probability of terminating head casts earlier in their outward swing. This leads to shallower turns, and therefore less tight localisation around the source. Increasing the scaling on the weathervaning kernel does slightly increase performance, although still not to the level of run or cast termination alone.

While run termination alone can reproduce larva-like levels of chemotaxis, we unsurprisingly see that there are mismatches between all the ‘single-mechanism’ variants of the model and the larva when we look at the low level statistics of behaviour—for example the model using only run termination fails to show a biasing of turn directions of a magnitude comparable to the larva (S2b Fig).

### Effects of noise on chemotaxis

Finally, we explored the performance of the model in a selection of distinctly different odour landscapes (linear, Gaussian, and step), with different amounts of noise, and with different combinations of the three behavioural biases. This provides a useful parallel to the analysis in [25] for a model of *C. elegans*. We analyse each larva’s performance with a Chemotaxis Index (CI); each larva is assigned a CI equal to the proportion of time spent in a region of interest (ROI); note that due to the differences in ROI areas, direct comparisons of performance between conditions cannot be made. The gradients used were all contained within a 9cm diameter circular arena, with parameters as follows. **Linear:** Concentration varying linearly from 0 at leftmost edge to 1 at rightmost edge. Region of interest is the rightmost 30mm of dish. **Gaussian:** Gaussian odour distribution, centred at the centre of the dish, with peak concentration 1 and variance 16mm. Region of interest is a 25mm diameter circle around the centre of the dish. **Step:** Odour concentration of 0 on left side of the dish and 1 on right, with 5mm wide linear transition of concentration between the halves. Region of interest is the right half of the dish.

**Fig 8:**
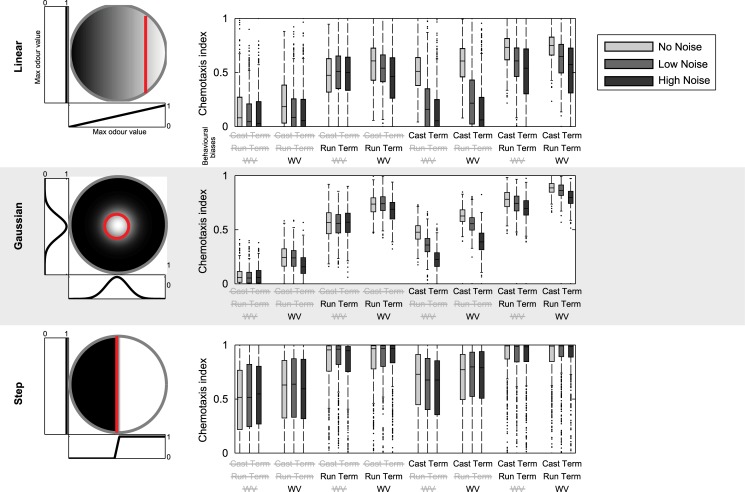
Chemotaxis indices in different simulated gradients. Performance of 500 simulated larvae in three different odour environments (displayed on the left), each with an appropriately defined region of interest (to the right of / inside the red line). Boxplots on the right show chemotaxis indices (proportion of time spent in region of interest) for each environment. Each combination of model mechanisms is run with three different levels of multiplicative noise.

For each combination of mechanisms (as in Fig 7) we ran 500 simulated larvae in each of the three environments, starting at a random initial position and orientation. This was repeated with multiplicative noise added to the environment by dividing the arena into an 0.08mm square grid and multiplying the concentration in each square by a value picked from a normal distribution with mean 1 and variance 0.04 (low noise) or 0.1 (high noise). CIs for each condition are shown in Fig 8.

The results suggest that some mechanisms may make smaller or greater contributions depending on the conditions. For example, including all three mechanisms seems to make a difference for the Gaussian gradient but not the other conditions, where weathervaning makes little contribution. CIs in the linear gradient are more affected by noise than the other conditions. The run termination mechanism seems more effective than the cast termination in the step gradient, and in general the contribution of cast termination is more affected by noise.

## Discussion

Our aim in this paper was to implement a minimally complex model that captures the observed odour taxis behaviour of *Drosophila* larvae. We show that larva-like behaviour can be achieved using relative change of odour concentration at the tip of the head combined with simple linear kernels to trigger transitions between behavioural states. Tuning the parameters of this model to match the detailed observations of larvae reported in [19, 21] produces behaviour that also matches the larva on other measures (such as proportions of head casts, under-correction of the heading angle, behaviour on different odour gradients). However, to reproduce typical preference indices reported for en masse assays we needed to alter the parameters to substantial weaken the effects of odour concentration on state transitions. This is discussed further below.

Our model combined three mechanisms, acting to modulate the probability of state transitions between running and head casting. In the absence of odour, the simulated larvae exhibit exploratory behaviour, making runs with regular small ‘weathervane’ head casts that are paused on random occasions leading to shallow curves, and also randomly stopping the run and making larger head casts, with random transition back to running, which can produce sharp turns. If this behaviour occurs in an odour gradient, the probability of stopping a run is enhanced for decreases and suppressed for increases in the change in odour concentration. The probability of restarting a run is enhanced by sharp increases in odour concentration during head casting. Small head casts during runs are also paused more often when the experienced odour concentration increase is greater than that already occurring in the run, resulting in a ‘weathervane’ curve towards the odour. From our simulations, it appears that either of the first two mechanisms would be sufficient to get the larva to an odour in a smooth gradient. Combining both with weathervaning produces the best performance; the improvement is most apparent in a Gaussian distribution, which is perhaps closest to the expected gradient for a point source of odour. Run termination is more robust to noise, which might be expected as (at least in our implementation) it averages input over a longer time scale than the other mechanisms.

The first mechanism (alteration in the rate of transitioning from running to head casting depending on the change in concentration) is equivalent to bacterial klinokinesis or the modulation of pirouette frequency observed in *C. elegans* [26] and is well-known to be sufficient to ascend a gradient. The second mechanism (ending head casting and resuming running when the head cast produces a sharp change in concentration) is klinotaxis, and we have shown it is also sufficient for chemotaxis on its own, producing similar performance to pure klinokinesis (when the latter is tuned to match larval behaviour). The combination of these two mechanisms substantially improves the efficiency of odour localisation over either alone. However, pure klinokinesis can potentially produce better chemotaxis if the parameters are tuned to optimise its performance. The final mechanism, weathervaning, is seen to make marginal improvements to chemotaxis performance, although it does not lead to robust chemotaxis without additional biases in the other mechanisms. It has been hypothesised that biasing of larvae’s run curvature is facilitated by low amplitude head casts made while running [21]. Our model concretely implements this hypothesis by including continuous low-amplitude head casting, which is temporarily paused by increases in the perceptual signal. We demonstrate that this mechanism can produce biases in run curvature comparable to that of the real larva, but other mechanisms, such alteration in the size of these casts, are also possible.

[19] show that larvae’s first head casts after terminating runs tend to be in the direction of higher odour concentration. We suggest that this is possible due to the larva having information about the lateral gradient from weathervaning during its run, and show that a similar level of first headcast bias can be produced if the simulated larva simply casts in the direction of its current head angle when terminating a run. This interaction of weathervaning and cast direction accords with the observation that the direction of run curvatures and subsequent turns are correlated [21].

Although treated here as a distinct mechanisms, klinotaxis and weathervaning could be interpreted as the same underlying orientation algorithm, i.e., exploiting the lateral sweep of the head through the gradient to obtain information about the odour direction, and altering the timing or extent of the sweep to orient the animal up the gradient. In the case of *C. elegans*, their oscillatory forward locomotion naturally produces a substantial sweep (relative to body length) and can be altered to produce relatively tight curves. In larvae, the peristaltic propulsion during runs appears to be inconsistent with large head casts, so while biasing the production of small head casts can steer the animal up the gradient, direct approach is only possible by stopping to make larger casts (or indeed, it may be that making a large cast forces a stop). It remains to be discovered how independent are the neural mechanisms underlying these behaviours in the larva.

The majority of behavioural experiments on larvae report only preference indices (PI), i.e., a binary classification of larvae as either within or without a designated region defined in relation to the stimulus of interest. It is important to understand how such global measures relate to the underlying behavioural control if the neural circuits involved in innate and learned sensorimotor control are to be explained. An issue revealed by our analysis is the difficulty of interpreting behavioural statistics that are derived by summing over many individuals, and over relatively long time durations. It was necessary to make each of the behavioural biases around 20 times weaker in a simulated larva to obtain ‘typical’ PIs. The discrepancy between the level of behavioural bias required to match PI data and the low-level behavioural statistics reported in [19, 21] could have multiple sources: different larvae may have different innate capabilities or preferences for particular odour sources; the attraction of an individual larva to an odour source may change over time due to habituation or changing motivational state or competition from other cues; or the odour gradient itself may vary substantially in the reliability with which it corresponds to the actual odour direction, both over time and space, in a typical Petri-dish experiment. It is clear that this issue can only be resolved by studies that track individuals over time in well-controlled or measurable stimulus conditions.

It is important to note that both previous biological experiments [16] and our simulations indicate that the larva can locate odours with a single point sensor on its head, and does not need spatially separated sensors, even for weathervaning. Rather, gradient information is gained through stereotypical movements over time. Nevertheless it is clear that the perceptual response to the odour gradient used in the simulation, which performs perfect differentiation and normalisation, is not realistic. In the majority of the behaviour analysed here the larva spends a large proportion of its time close to the odour peak, and thus in a relatively limited range of concentrations. As such, normalisation should not have a large impact on the model’s global behavioural statistics. However, it is likely to play a significant part in the model’s ability to localise the peak from a distance, by making the simulated larvae unrealistically sensitive to small differences in areas of low odour concentration. In future work, it will be interesting to incorporate more detailed olfactory receptor responses (such as described in [27]) into the model, and see how these interact with both different odour gradients and the dynamics of the motor actions to shape the overall behaviour, particularly in relation to approaching an odour from a distance.

We have also used a highly simplified model of the larva’s motor system. Although ‘runs’ and ‘head casts’ are reasonable approximations to the main observable actions by the larva, further analysis may reveal important subtleties. For example, the peristaltic pattern that produces the run also imposes a pattern on the sensory input, as the head moves forward and pauses on each cycle, and also changes its orientation with respect to the substrate. Similarly, the rather arbitrary distinction between ‘small’ and ‘large’ head casts used in the simulation may need more detailed representation of the form, size and location of body bends of which the larva is capable.

Finally it may be interesting to ask whether a simpler control scheme than the state transitions illustrated in Fig 1b might give rise to qualitatively similar behaviour. It is interesting to note that the mechanisms used to produce the different behavioural transitions in our model are all fundamentally the same, involving differentiation, integration and a non-linear switch, and differ only in their timescales and their weighting of the perceptual signal. Should we assume the current characterisation will map onto distinct ‘decision’ circuits in the animal for changing between runs and head casts? Or is it possible that these are emergent properties of lower level control that integrate the muscle contractions producing both peristalsis and body bends and modulates them in response to sensory input?

## Supporting Information

S1 Fig Behavioural statistics for the simulated larva with no weathervaning bias. Produced following the same procedure as Fig 2. With the weathervaning bias removed from the model, the bias of run reorientation (row 3) towards the odour is lost.
(PDF)

S2 Fig Single mechanism performance with optimised kernel scaling. All results were obtained by collecting statistics from simulated larvae in the single odour source environment, as described for Figs 2 and 3. a) Mean and standard error of distance to the odour peak after 120s for 200 larvae with only one behavioural bias, plotted against a range of kernel scaling values. We used these plots to determine the approximate kernel scaling which gave the closest clustering around the source for each behaviour bias; scaling = 200 for run termination, scaling = 1 for cast termination, and scaling = 20 for weathervaning. b) Low level behavioural statistics produced by each of the three single-bias model variants when the kernel is scaled by the value determined in a). Behavioural statistics from the larva and the full model are included for comparison. Note that the single-mechanism models fail to match the larvas statistics as well as the full model, both due to the lack of the other mechanisms (e.g. the run-termination only model shows low bias in left turn probabilities due to the lack of cast-termination bias) and the excessive strength of the scaled bias (e.g. the run-termination only model shows a higher rate of runs of length 0–10s due to high scaling of the run termination kernel).
(PDF)
