# Supplementary material for: A Model of Drosophila Larva Chemotaxis
Source: PLoS Comput Biol. 2015 Nov 24;11(11):e1004606. doi: 10.1371/journal.pcbi.1004606 (PMC4657977; doi:10.1371/journal.pcbi.1004606)
Supplement: S1 Fig — Produced following the same procedure as Fig 2. With the weathervaning bias removed from the model, the bias of run reorientation (row 3) towards the odour is lost. (PDF) [file pcbi.1004606.s001.pdf]

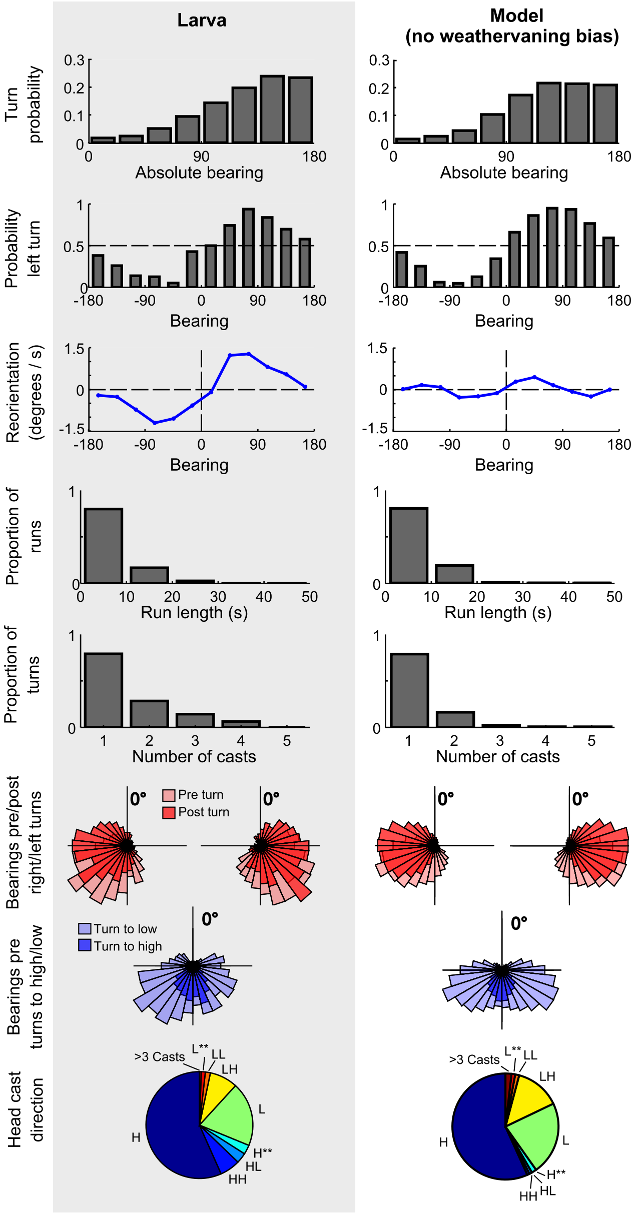

Behavioural statistics for the simulated larva with no weathervaning bias. Produced following the same procedure as figure 2. With the weathervaning bias removed from the model, the bias of run reorientation (row 3) towards the odour is lost.
