# Supplementary material for: A Model of Drosophila Larva Chemotaxis
Source: PLoS Comput Biol. 2015 Nov 24;11(11):e1004606. doi: 10.1371/journal.pcbi.1004606 (PMC4657977; doi:10.1371/journal.pcbi.1004606)
Supplement: S2 Fig — All results were obtained by collecting statistics from simulated larvae in the single odour source environment, as described for Figs 2 and 3. a) Mean and standard error of distance to the odour peak after 120s for 200 larvae with only one behavioural bias, plotted against a range of kernel scaling values. We used these plots to determine the approximate kernel scaling which gave the closest clustering around the source for each behaviour bias; scaling = 200 for run termination, scaling = 1 for cast termination, and scaling = 20 for weathervaning. b) Low level behavioural statistics produced by each of the three single-bias model variants when the kernel is scaled by the value determined in a). Behavioural statistics from the larva and the full model are included for comparison. Note that the single-mechanism models fail to match the larvas statistics as well as the full model, both due to the lack of the other mechanisms (e.g. the run-termination only model shows low bias in left turn probabilities due to the lack of cast-termination bias) and the excessive strength of the scaled bias (e.g. the run-termination only model shows a higher rate of runs of length 0–10s due to high scaling of the run termination kernel). (PDF) [file pcbi.1004606.s002.pdf]

**Supplementary figure 1**

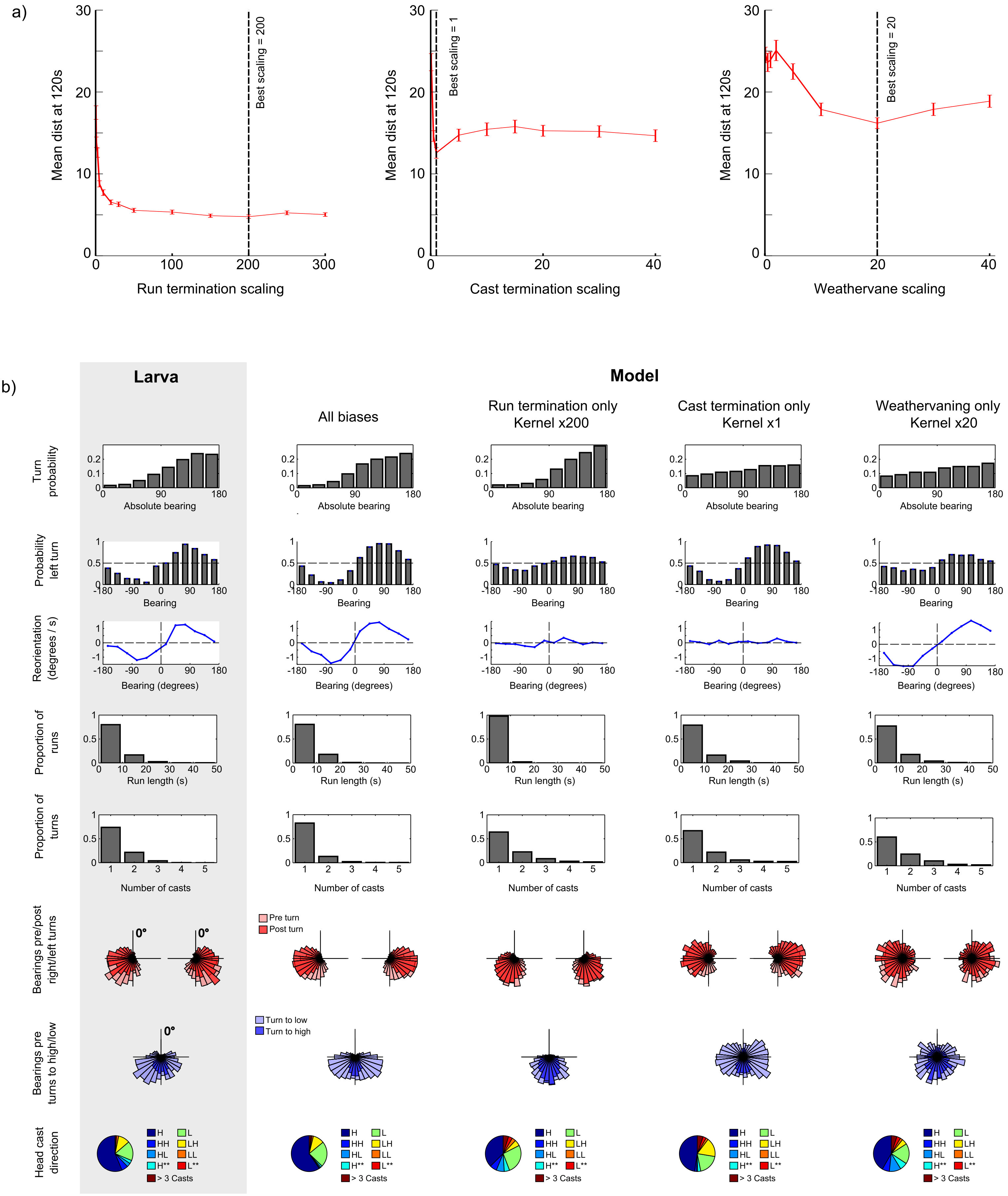


**Single mechanism performance with optimised kernel scaling.** All results were obtained by collecting statistics from simulated larvae in the single odour source environment, as described for figures 2 and 3. **a)** Mean and standard error of distance to the odour peak after 120s for 200 larvae with only one behavioural bias, plotted against a range of kernel scaling values. We used these plots to determine the approximate kernel scaling which gave the closest clustering around the source for each behaviour bias; scaling = 200 for run termination, scaling = 1 for cast termination, and scaling = 20 for weathervaning. **b)** Low level behavioural statistics produced by each of the three ‘single-bias’ model variants when the kernel is scaled by the value determined in a). Behavioural statistics from the larva and the full model are included for comparison. Note that the ‘single-mechanism’ models fail to match the larva’s statistics as well as the full model, both due to the lack of the other mechanisms (e.g. the ‘run-termination only model’ shows low bias in left turn probabilities due to the lack of cast-termination bias) and the excessive strength of the scaled bias (e.g. the ‘run-termination only’ model shows a higher rate of runs of length 0-10s due to high scaling of the run termination kernel).
